# Career Ambition as a Way of Understanding the Relation Between Locus of Control and Self-Perceived Employability Among Psychology Students

**DOI:** 10.3389/fpsyg.2018.01729

**Published:** 2018-09-19

**Authors:** Maja Ćurić Dražić, Ivana B. Petrović, Milica Vukelić

**Affiliations:** ^1^Department of Psychology, Faculty of Media and Communications, Singidunum University, Belgrade, Serbia; ^2^Department of Psychology, Faculty of Philosophy, University of Belgrade, Belgrade, Serbia

**Keywords:** students’ self-perceived employability, locus of control, career ambition, Serbia, psychology students, future work skills

## Abstract

Employability is one of the core concepts for the future career. Students’ self-perceived employability is the concept that connects students’ present context of education with their future professional engagement. Students’ self-perceived employability is defined as the capacity to gain and keep employment in line with their future qualification level. Locus of control is a concept that explains where the person situates the causation of various life events. It is found that internal locus of control was related to different aspects of career success. Career ambition is regarded as a proxy for students’ future career success. Both internal locus of control and ambition lead to proactive behaviors that are relevant for employability and consequently result in securing a sustainable job. The aim of this mixed-method study was to investigate the relations among locus of control, ambition and students’ self-perceived employability. Firstly, we tested mediating role of career ambition in relation of internal locus of control and students’ self-perceived employability, then we turned to qualitative analysis of students’ career self-SWOT analyses in order to deepen and enrich quantitative findings. The sample consisted of 124 undergraduate psychology students that filled out Levenson’s Internality subscale from IPC locus of control scale, Rothwell et al. (2008) Ambition subscale, and three items extracted from the Self-perceived employability subscale. Majority of the survey participants (*N* = 100) filled out personal career SWOT analysis. The mediation analysis showed that career ambition had a mediating role in the relation between the locus of control and employability. Students perceived personal capabilities and ambition as internal strengths and lack of ambition as a major internal weakness. As external opportunities students perceived various chances for developing professional skills, whereas as external threats they perceived limited opportunities in job market. In order to support university students to develop employability and future career success, university curricula should support developing future work skills that, in addition to functional competences and personal resources, entail career ambition, ways of utilizing external opportunities and dealing with job market threats.

## Introduction

In the fast changing socio-economical context traditional career has been gradually leaving the stage and letting the place to modern, dynamic, flexible, fragmented, boundaryless and jobless career (Petrović and Čizmić, 2015). In this context employability has been attracting the attention from scientific and research perspective that partly lead to policy making, and partly to practical interventions (Bernston, 2008; Vanhercke et al., 2014). At its simplest, employability deals with the ability to be employed (e.g., Hillage and Pollard, 1998; Vanhercke et al., 2014). From the scientific literature based on the number of conceptualizations and operationalizations, it is evident that approach to employability differs across disciplines (Forrier and Sels, 2003).

In psychological approach, employability is mainly defined as set of competences or as a set of dispositions (De Cuyper et al., 2012). The competence-based approach to employability deals with persons’ perception of abilities, capacities and skills that are the bases of employment (Van der Heijde and Van der Heijden, 2006). Employability as a competence could be regarded as the first step toward employment as such. It is of a particular interest for students on one side, and educational institutions or providers of education for labor market needs, on the other (Hillage and Pollard, 1998; Harvey, 2001; Knight and Yorke, 2004). Dispositional approach focuses on the perception of person’s proactive attitudes related to the career and work in general. This approach was particularly developed by Fugate and Kinicki (2008, p. 504) who conceptualized employability as “a constellation of individual differences that predispose employees to (pro)actively adapt to their work and career environments.”

In the overall context of constant change of labor market conditions, one’s perceptions of his/her personal employability became increasingly important (Bernston, 2008). Perception of employability as personal appraisal of possibilities of getting the first or new employment is significant because it affects persons’ overall well-being (Bernston, 2008; Vanhercke et al., 2014; Giorgi et al., 2015). Even though the concept of perceived employability is of high interest for those who work, it is also important to investigate employability among those who enter the job market, i.e., students. It is widely recognized that youth un/employment, moreover graduate un/employment, is among the pressing issues of youth well-being (International Labour Office, 2017). Strikingly enough, there are only a few research studies directly dealing with students’ employability (e.g., Rothwell et al., 2008; Dacre Pool et al., 2014). Although there are no studies of students’ employability, there are certain studies that deal with different forms of career behaviors as well as personal characteristics that could be related to the employability. For instance, Kuijpers et al. (2011) and Kuijpers and Meijers (2012) point out the importance of career learning in education, which aims to develop career competences. Career competences may develop through four-factor model of career competences that includes career reflection, work exploration, career action and networking. Developing these competences among students may consequently enhance students’ employability. Further, findings from the study of Zellweger et al. (2011), indirectly indicate that entrepreneurial career, as a one of the ways of getting the job in accordance with personal interests, may also improve students’ employability.

In support of the fact that students’ employability is an important issue, statistics show that in EU in 2016 more than 6.3 million young people aged 15–24 years were neither employed nor in the process of educating or training (European Commission, 2018). The situation is similar in Serbia. After the last three decades of struggling with long-lasting socioeconomic crisis, firstly influenced by political instability in 1990s, and then by the global financial crisis (Petrović et al., 2017), low economic activity in Serbia is characterized by high unemployment rate of 14.1% among population aged 15–64 and among those of 15–24 years of age that percentage rises up to 31.9% (Statistical Office of the Republic of Serbia, 2017).

Psychology education in Serbia is marked by the Law on Higher Education (Zakon o visokom obrazovanju, 2017) that regulates adhering of higher education institutions to the Bologna process and joining European Higher Education Area (EHEA) on one side, and regulation of psychology profession and European Diploma in Psychology (Lunt et al., 2015), on the other. All accredited psychology university programs in Serbia give opportunities to students to go out of the university to get employment after having 180, 240, or 300 ECTS. However, for the professional practice of all fields of psychology it is necessary to complete psychology master program equivalent to 300 ECTS. Based on previous research about students’ career related needs, psychology programs were modified to better answer the changing needs of students in preparing them for the job market and transition to work not just by securing better employability, but also by equipping them for more successful behavior on the job market (Petrovic et al., 2010, 2011).

Locus of control (LOC) is a concept that explains where the person situates the causation of various life events (Rotter, 1966). Persons with internal LOC believe that they have control over their lives, whereas those with external locus believe that their lives are largely controlled by external outside factors. Researchers found that internal LOC was related to better college adjustment (Aspelmeier et al., 2012), as well as to different aspects of career success. LOC was found to be associated with job-related behaviors such as job satisfaction (Judge and Bono, 2001), performance, greater effort at work, self-efficacy and career success (e.g., Ng et al., 2006). Research in Serbia has shown that work LOC (beliefs about the source of control at the workplace) was positively related with proactive job search (Petrović et al., 2009). Theoretically speaking, different authors suggest the connection between internal LOC and behaviors such as need for achievement, perceptions of opportunities at work and work motivation that could be explained by person’s beliefs about the existence of link between efforts and outcomes (Patton et al., 2004; Ng et al., 2006). Thus, exerting greater effort that stems from the internal LOC can be perceived as a source of striving for achievement.

H1: Locus of control relates positively to employability.

Ambition could be regarded as an underlying mechanism that puts values on outcomes, connecting beliefs about personal efforts that stem from internal LOC to career success (Rothwell et al., 2009). Based on the research of Rothwell et al. (2009), we state that in population of students ambition could be regarded as twofold mechanism – on one side it is a more general indicator of future professional success, and on the other side, exerting effort in successful studying closely advances employability. Undoubtedly, all three concepts shed light on dealing effectively with situations that lead to successful employment. Based on the following we state next three hypotheses.

H2: Locus of control relates positively to career ambition.
H3: Career ambition relates positively to employability.
H4: Relationship between locus of control and students’ self-perceived employability is mediated by their career ambition.

In a nutshell, the aim of this study was to investigate the relations among LOC, ambition and students’ self-perceived employability. Firstly, we tested mediating role of career ambition in relation of internal LOC and students’ self-perceived employability, then we turned to qualitative analysis of students’ career self-SWOT analyses in order to deepen quantitative findings.

Career self-SWOT analysis is the application of SWOT analysis in the career planning process. SWOT analysis is a widely used business tool for situation analysis in the process of organizational strategy planning (Ghazinoory et al., 2011). Acronym stands for assessing organization’s strengths, weaknesses, opportunities, and threats (e.g., Kotler and Armstrong, 2017). Organizational strengths include internal capabilities and resources that may support the organization in achieving its strategic aims and goals, whereas weaknesses are organization’s internal limitations that may obstruct realizing its aims and goals. Opportunities and threats are considerations, conditions, trends or aspects of organization’s external environment that could be advantageous or unsupportive of organization’s performance.

Initially developed for securing the success of corporate planning (Chapman, 2004; Friesner, 2018), due to its usefulness and “ability to flow” (Røvik, 2002), SWOT analysis spread easily beyond companies to not for profit business, countries and industries (Helms and Nixon, 2010) and project planning in general. Career planning is one of the areas where we can find promising application of SWOT analysis (e.g., Addams and Allfred, 2013). It comes as a natural step in the process of learning and practicing application of business tools by students that take some of the business courses (e.g., McCorkle et al., 2003), but it can equally well be applied in career preparation of students from other fields. Just as it was developed to secure the success of companies’ strategic planning, it can be expected to support students’ career development and success.

Even though the graduate unemployment is an acknowledged international problem (International Labour Office, 2017), there is not enough research about students’ perceptions of their own employability in general, as well as those that explore underlying mechanisms that could explain perceived employability and lead to sustainable intervention. The study could be of a particular importance for exploring and understanding the psychological dynamic of students’ perceived employability. Both LOC and career ambition are interesting in the field of intervention, particularly in economies and labor markets that are strongly affected by instability and global economic crisis (Mucci et al., 2016; Lopez-Valcarcel and Barber, 2017). Under these circumstances personal beliefs about the source of the control over life along with career ambition can be essential for finding, sustaining or creating job opportunities. Since the concept of perceived employability among students has not been extensively investigated, both quantitative and qualitative approach were chosen for securing more thoroughly founded results.

## Materials and Methods

### Participants

This mix-method study is based on two kinds of data initially collected for educational purposes. Quantitative data were based on a survey that covered a sample of 124 psychology students (89.1% female) from the third and fourth year of 4-year undergraduate studies from a university in Serbia. Majority of participants were younger than 23 years (73.2%). Participation in the survey was anonymous, voluntary and not compensated. The study was carried out in accordance with the Code of Ethics of the Serbian Psychological Society, and approved by the Committee on Ethical Issues of the Society of Psychologists of Serbia Ethics Commission at the Department of Psychology, Faculty of Philosophy, University of Belgrade, with written informed consent from all participants. Materials for the qualitative analysis consisted of students’ personal career SWOT analyses in which they analyzed career related strengths, weaknesses, opportunities, and threats on the labor market. This was the personal reflection assignment for the Career management course. There were 100 students that both participated in the survey and handed in SWOT analyses. Even though students personally handed their SWOT analyses as the course activity, assignments were anonymized for this research.

### Instruments and Procedure

#### Career Self-SWOT Analysis

Career self-SWOT analysis as the application of SWOT analysis in the career planning process was a self-reflection assignment in which students had to analyze and assess their personal strengths and weaknesses; opportunities and threats from the external environment, all in relation to career. Students were firstly informed about the technique, and it was stressed that they have the full freedom to fill the SWOT grid according to their personal evaluation of their overall career situation. The students were encouraged to list at least three features in each of the SWOT fields and majority of students listed exactly the required number of features in every SWOT field.

#### Career Ambition

Career ambition was assessed by the *Ambition subscale* from the *Self-perceived employability scale* (Rothwell et al., 2008). The career ambition subscale consists of 6-items that are rated on 5-point Likert scale between 1, indicating complete disagreement and 5, indicating complete agreement. The examples of items are: “*I have clear goals for what I want to achieve in life*” and “*I feel it is urgent that I get on with my career development.*” Rothwell et al. (2008) reported Cronbach’s alpha of 0.66. In this research, Cronbach’s alpha for Ambition subscale was 0.60. Alpha if item deleted analysis showed that the item 6 significantly decreased reliability in our sample (“*What I do in the future is not really important*”). All three authors agreed that there was an issue with the content validity of this item since it was not clearly related to ambition. After excluding this item from the subscale, the reliability of the Cronbach’s alpha raised to 0.76. Thus, we have used five items instead of original six items in further analyses.

#### Locus of Control

Locus of control was assessed by the *Internality subscale* from the *Levenson’s IPC Scale* (Levenson, 1973). The examples of items are: “*When I make plans, I am almost certain to make them work*”; *“When I get what I want, it is usually because I worked hard for it.*” The Levenson’s subscale consists of eight items that are rated on a 4-point Likert scale from 1, indicating complete disagreement, to 4, indicating complete agreement. Since the results of the reliability analysis of the Serbian adaptation of the subscale administered in this research showed that two items (items 4 “*Whether or not I get in to a car accident depends mostly on how good of a driver I am*” and 9 “*How many friends I have depends on how nice a person I am*”) decreased reliability (Cronbach’s alpha for the whole subscale was 0.58, and Cronbach’s alpha without these two items was 0.70), in further analyses we used the Levenson’s subscale without these two items, i.e., with six items.

#### Self-Perceived Employability

Self-perceived employability was assessed by the three items from the *Internal employability subscale* from the *Self-perceived employability scale* (Rothwell et al., 2008). The three chosen items were: “*The skills and abilities that I possess are what employers are looking for*”; “*I am generally confident of success in job Interviews and selection events*”; “*I feel I could get any job so long as my skills and experience are reasonably relevant.*” The reliability of these three items measured by the Cronbach’s alpha was 0.79. Each item was rated on a 5-point Likert scale from 1, indicating complete disagreement, to 5, indicating complete agreement.

All of the subscales used in the present study were translated to Serbian through the committee technique and translations were checked applying back translation (Brislin et al., 1973). Committee translation was carried out in two iterations (independent parallel translation and collaborative developing of consensus-based Serbian versions of subscales). Authors of the paper were committee members. Translated subscales were back translated into English by an independent bilingual researcher. Back translations were checked for consistency with the originating English versions by the committee and independent researcher that did back translation. After confirming the Serbian translation adequacy, we included the translated subscales in the questionnaire.

## Results

Before the main mediation analysis, we previously tested whether a single factor explained the majority of covariance among the instruments applied in this study. The results of the Herman’s single factor test using one factor un-rotated factor analysis showed that 34.6% of a total variance was explained. This means that the common method variance could not explain the larger part of the covariance between items from the applied instruments.

In order to explore whether each item loaded accurately on the supposed component, we performed additional factor analysis. Further analysis has shown that each item loaded accurately on assumed component, all loadings were higher than 0.50, except for the two items from Internality subscale from the Levenson’s IPC Scale (Levenson, 1973), that were a little lower, i.e., 0.46 and 0.43, but positioned on the presumed component (“*When I get what I want, it is usually because I worked hard for it*,” “*When I make plans, I am almost certain to make them work*”). Since these items did not decrease the reliability of the Internality subscale, we retained them and further analyses were done with six items.

The means, standard deviations, skewness and kurtosis values, and correlations among internal LOC, ambition and employability are presented in **Table 1**. As it can be seen from the **Table 1**, LOC, ambition and employability are positively and moderately correlated. The values of skewness and kurtosis for the Ambition subscale point to the fact that in this sample we have negatively skewed, leptokurtic distribution. It is reasonable to expect such distribution as the sample comprised of successful students that had already passed 2 or 3 years of a demanding undergraduate program.

**Table 1:** Means, standard deviations, skewness, kurtosis and correlations among internal locus of control, ambition and employability.

	*M*	*SD*	Skewness (*SE*)	Kurtosis (*SE*)	Ambition	Employability
Internal locus of control	20.12	2.23	0.020 (0.220)	−0.726 (0.437)	0.517^∗∗^	0.438^∗∗^
Ambition	21.94	2.62	−1.365 (0.218)	2.382 (0.433)		0.538^∗∗^
Employability	11.35	2.03	−0.146 (0.217)	−0.102 (0.431)		

*^∗∗^Correlation is significant at the 0.01 level.*

**FIGURE 1:**
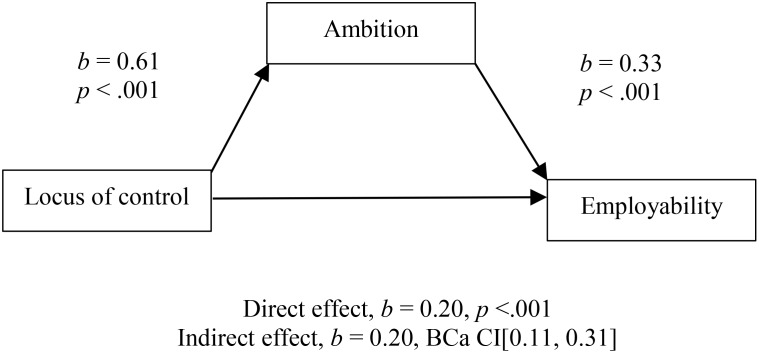
Model of internal locus of control as a predictor of employability mediated by career ambition.

In order to test presented hypotheses, we have performed mediation analysis. Mediation analysis (see **Figure 1**) has shown that there was a significant indirect effect (Sobel test, *z* = 3.82, *p* = 0.00) of internal LOC on employability through the career ambition (*b* = 0.20, BCa CI [0.11, 0.31]). In addition, it is worth mentioning that multiple regression analysis has shown that two predictors explained 32% of the variance [*R*^2^ = 0.32, *F*(2,118) = 28.28]. It is also of importance to note that the multicollinearity assumption has not been violated, as can be seen from the VIF = 1.36 and Tolerance = 0.73 (**Figure 1**).

The career self-SWOT analysis features that students listed as their personal strengths and weaknesses, and external opportunities and threats are presented in **Table 2**. Qualitative analysis is based on career self-SWOT analyses developed by 100 students. It should be noted that students had a problem to list more features than the required minimum of three that led to a more simple SWOT than could have been expected. We organized listed features into categories that are also presented in **Table 2**. Abilities were listed only as a personal strength. As initially SWOT analysis covers organization’s internal and external environment, career self-SWOT analysis, perceived in the context of the LOC, covers personal strengths and weaknesses as connected with internal locus and opportunities and threats as related with the external locus.

**Table 2:** Career self-SWOT analysis: factors organized by categories and their frequencies.

Strengths	Weaknesses
***Abilities***	
Intelligence (21)^∗^	
Creativity (21)	
Readiness to learn (15)	
***Motivation and other characteristics***	
Proactivity (36)	Lack of motivation (36)
Ambitiousness (33)	Lack of self-confidence (36)
Perseverance (30)	Low efficiency (33)
Responsibility (27)	Procrastination (33)
Initiative (18)	Lack of determination (27)
Commitment (15)	Lack of self-discipline (21)
Resourcefulness (15)	Lack of organization (21)
Adaptability (12)	Laziness (21)
Thoughtfulness (12)	Too much relying on authorities (15)
**Opportunities**	**Threats**
***Professional networking***	***Weaker position of the faculty and university***
Developing contacts with psychologists from the practice (18)	Prejudices about private universities (33)
Developing contacts in the academic community (18)	
Collaborating with university teachers (15)	
***Opportunities for employment***	
Growing number of organizations that employ psychologists (27)	Lack of job openings (57)
Contemporary society’s needs for psychological services (24)	Instability of economy (36)
Private psychotherapy practice (24)	Limited employment in the public sector (21)
Development and better opportunities for the funding of civil service organizations (18)	
Growing social and economic support for entrepreneurship (18)	
***Developing of professional competencies and practical experience***	***Job market competition***
Internship (39)	Growing numbers of graduated psychologists (57)
Skills training (36)	Higher unemployment rate among young people (12)
Professional volunteering (21)	
	***Irregularities in hiring***
	Nepotism (21)
	Hiring based on connections (12)

*^∗^The number of students who listed the feature.*

However, it should be noted that features related with ambition have been rather richly presented, both as a personal strength and as a personal weakness. There is more diversity in listed strengths than weaknesses. Altogether, listed personal strengths and weaknesses clearly point that students perceive work performance features that could be subsumed under the concept of ambition as important for their future career. As for the external factors of students’ future career, it could be noted that opportunities are more tied with “important others” (mainly university professors and internship mentors), whereas threats are more tied with more distant market and economy conditions in the country (such as high unemployment rate, low economic activity). It is interesting to note that these findings are in the line with the previous research findings from Serbia in which initial factor of external control from the Spector’s Work locus of control scale (Spector, 1988) was divided in two factors – external locus defined by the social domain, labeled as “powerful others” (Blakely et al., 2005), and external locus defined by luck and external circumstances that corresponded to factor of luck from the Blakely et al. (2005) research.

## Discussion

It is recognized in the literature that employability plays an important role in securing actual employment of people with previous work experience (King, 2004). In the population of students it is yet needed to explore the concept of employability in depth, the ways it is connected with various students’ job search and work-related outcomes, as well as the way of its operationalizing. For university students, it is particularly important to get to know personal resources (Fugate et al., 2004), characteristics and competences that lead to better self-perceived employability as well as to actual employment. LOC is one of these personal resources that had been recognized as a significant correlate of job-related behaviors and career success (Judge and Bono, 2001; Petrović et al., 2009). Internal LOC, besides being a good predictor of academic success and college adjustment (Aspelmeier et al., 2012), may also lead to the consideration of self-employment and entrepreneurial career (Zellweger et al., 2011) which could be connected with better employability of students. The core of LOC is the perception of control over the significant life events which is firmly connected with the determination to achieve success. Even though this connection could be regarded as obvious and intuitive, research that explored and explicated the nature of the connection between LOC and career success obviously lacks.

Studying relationships of LOC, ambition and self-perceived employability in the sample of third and fourth year students of 4-year undergraduate psychology program, applying the mediation analysis we found that career ambition had a mediating role in the relation between LOC and employability. LOC is strongly and positively correlated with ambition and employability, while ambition is strongly and positively related to employability (see **Figure 1**). This research has shown that, in the population of university students, career ambition could be regarded as an underlying mechanism that connects LOC and employability. In the population of students at different levels of education, in which researchers cannot use widely known measures of career success, ambition could be observed as a proxy for future career success. This research also supports the idea that students’ ambition can be regarded as the glue that connects career-related dispositions and future career-related achievements.

Digging deeper into the relationships among students’ dispositions and their perceptions of various personal and contextual aspects of career success, we turned to analyzing their personal career-related SWOT reflections. Based on the students’ personal career-SWOT analyses, it is evident that attributes that could be considered as the indicators of the lack of ambition were mostly considered as personal weaknesses (**Table 2**). Within weaknesses part of the grid the attributes such as –low self-efficacy, procrastination, low self-confidence, low self-esteem, could be found. On the other hand, leading strengths are ambition, proactivity, flexibility, perseverance, and alike. Opportunities and threats usually come from the broader social context which could be only partially be addressed by personal strengths and career ambition. Still, it is evident from the career-SWOT grid that external features were elaborated more thoroughly than internal. It was evident that some potential, generally expected, internal strengths, as well as weaknesses, such as competences, skills, personality traits, knowledge were missing. It is questionable why students do not perceive these aspects as important for employability and future career. Presented findings clearly point out the importance of working with students on career related issues either through university course activities (functional competencies) or mentorship (career management competencies).

Although there are no directly comparable studies, results of the present study could be perceived as being in line with findings from studies which indirectly investigated students’ employability (e.g., Aspelmeier et al., 2012; Kuijpers and Meijers, 2012). Features obtained in SWOT analyses support the four-factor model of career competencies proposed by Kuijpers et al. (2011). In addition, results highlighted the importance of LOC for future career success which is in accord with findings from the study of Aspelmeier et al. (2012). The results of the present study are in agreement with the findings from the studies which emphasize the importance of learning environment and career dialog between students and important others (e.g., teachers, career counselors) for developing career related competencies (Kuijpers and Meijers, 2012), that could indirectly lead to improving students’ employability.

### Limitations and Implications for Future Research

Longitudinal study would have certainly been better suited for exploring students’ employability. Cross-sectional study in the case of students of final 2 years of 4-year undergraduate studies limits the generalizability of findings as studies itself lead to changes in self-evaluation in general, and self-perceived employability, in particular. At this point it is important to consider the limitations that stem from the sample size and its specificities. Choosing the psychology students of the final years, moreover from one university, could raise the question about obtained distributions of all three variables. However, the similar distributions can always be expected when exploring university students of final years of any study program. Even tough it would be valuable to explore employability of students of different professional fields, it was practically impossible to carry out mixed-method study as the present one due to lack of career related courses, even lack of interest for career related issues, different possibilities of getting a job at the labor market and alike. If such a sample was attainable it would have been extremely demanding to control all the relevant sources of variation to enable valid comparisons. Under such conditions, we opted for a coherent sample of two subsequent generations of psychology students that had Career management as one of the courses and had the same objective chances of getting employment after graduating in the near future.

As demonstrated with the reliability coefficients of applied subscales that were translated and adapted for use in Serbian, that were better than the reliabilities of the original subscales, but not ‘perfect,’ further work on refining the instruments and possibly developing better, more comprehensive, valid and reliable measures of all three concepts, i.e., career ambition, LOC and self-perceived employability is highly warranted. In addition, it should be noted that all the measures come from the same source. Adding external assessments of applied measures could significantly enhance the validity of the overall model. Teachers’ evaluations and longitudinal following of students’ employability on the course of their studies could enrich our understanding of employability in the population of students. The same goes for the ambition, where it would be helpful to correlate it with the objective behavioral indicators of students’ present ambition as well as with objective behavioral measures of future work performance that could be secured in a longitudinal research design. Last, but certainly not the least, it is widely acknowledged that researchers have not yet come up with a satisfactory measure of LOC. It is one of the concepts about which there is a high level of agreement about its importance, based on relevant consequences and outcomes. Despite of numerous attempts, there is still lack of widely accepted valid measure of LOC.

The applied mixed-method approach to exploring perceived employability among students enabled developing of a more rounded insight into the concept that could further open some new research routes. Presented findings call for further exploration of other personal resources that are connected with career success, as well as other career success indicators. Personal resources as self-esteem, self-confidence, and self-efficacy could further be considered (Dacre Pool and Sewell, 2007). Also, when it comes to future career success, other indicators such as career engagement could be taken into the consideration.

On the practical level, this study is of a particular importance in the context of exploring and understanding the psychological dynamic of students’ perceived employability that could be the inspiration for developing practical guidelines, mostly for those who work with students. One of the interesting practical findings come from the mismatch of perceived career ambition and competencies in the SWOT grids on one side, and negatively skewed distributions of scores on LOC, career ambition and self-perceived employability, on the other. Based on this kind of findings, all those that work with university students (such as teachers, career counselors, internship mentors), can develop interventions in their appropriate fields that would support students’ in developing competences, and, thus their self-perceived employability, as well as their career ambition.

## Conclusion

The unique finding that came from the presented research was that students’ career ambition had a mediating role in the relation between the LOC and self-perceived employability. Bearing in mind the scarce research literature on students’ self-perceived employability, this finding is even more valuable as it calls for attention to psychological dynamics of students’ employability. Students perceived personal capabilities and ambition as internal strengths and lack of ambition as a major internal weakness. As external opportunities students perceived various prospects for developing professional skills; as external threats they perceived limited opportunities in the job market. This research implies that developing and sustaining career ambition, as well as perceived control over career events, could lead to students’ perception of better employability.

As research comes from Serbia, country with a relatively high unemployment rate, it should be noted that some labor market circumstances limit considerably job search behaviors. Personal ambition and internal LOC are not omnipotent in the context of restricted job market opportunities. Likewise, we can expect that the global economic crisis can add a layer of understanding in the dynamics of perception of employability by students. The actuality and importance of the explored topic resonates far beyond the borders of Serbia, the country in which the research took place. The overall context of global economic crisis makes the issue of employability in general, and self-perceived employability, in particular, highly relevant for policy makers and all those who take part in creating economic conditions that enable higher employment rates and new jobs on the market.

## Author Contributions

MĆD, IP, and MV contributed equally to the research design and writing of this paper.

## Conflict of Interest Statement

The authors declare that the research was conducted in the absence of any commercial or financial relationships that could be construed as a potential conflict of interest.
